# A behavioral economic intervention to increase psychiatrist adherence to tobacco treatment guidelines: a provider-randomized study protocol

**DOI:** 10.1186/s43058-020-00011-x

**Published:** 2020-02-25

**Authors:** Erin S. Rogers, Christina Wysota, Judith J. Prochaska, Craig Tenner, Joanna Dognin, Binhuan Wang, Scott E. Sherman

**Affiliations:** 1 Department of Population Health, NYU School of Medicine, 180 Madison Avenue, New York, NY 10016 USA; 2 Department of Medicine, Stanford Prevention Research Center, Stanford University, 1265 Welch Road St, Stanford, CA 94305 USA; 3 VA NY Harbor Healthcare System, 423 East 23rd Street, New York, NY 10010 USA

**Keywords:** Tobacco use cessation, Psychiatry, Electronic medical record

## Abstract

**Background:**

People with a psychiatric diagnosis smoke at high rates, yet are rarely treated for tobacco use. Health care systems often use a “no treatment” default for tobacco, such that providers must actively choose (opt-in) to treat their patients who express interest in quitting. Default bias theory suggests that opt-in systems may reinforce the status quo to not treat tobacco use in psychiatry. We aim to conduct a pilot study testing an opt-out system for implementing a 3As (ask, advise, assist) tobacco treatment model in outpatient psychiatry.

**Methods:**

We will use a mixed-methods, cluster-randomized study design. We will implement a tobacco use clinical reminder for outpatient psychiatrists at the VA New York Harbor Healthcare System. Psychiatrists (*N* = 20) will be randomized 1:1 to one of the two groups: (1) opt-in treatment approach—psychiatrists will receive a reminder that encourages them to offer cessation medications and referral to cessation counseling; (2) opt-out treatment approach—psychiatrists will receive a clinical reminder that includes a standing cessation medication order and a referral to cessation counseling that will automatically generate unless the provider cancels. Prior to implementation of the reminders, we will hold a 1-h training on tobacco treatment for psychiatrists in both arms. We will use VA administrative data to calculate the study’s primary outcomes: (1) the percent of smokers prescribed a cessation medication and (2) the percent of smokers referred to counseling. During the intervention period, we will also conduct post-visit surveys with a cluster sample of 400 patients (20 per psychiatrist) to assess psychiatrist fidelity to the 3As approach and patient perceptions of the opt-out system. At 6 months, we will survey the clustered patient sample again to evaluate the study’s secondary outcomes: (1) patient use of cessation treatment in the prior 6 months and (2) self-reported 7-day abstinence at 6 months. At the end of the intervention period, we will conduct semi-structured interviews with 12–14 psychiatrists asking about their perceptions of the opt-out approach.

**Discussion:**

This study will produce important data on the potential of opt-out systems to overcome the barriers in implementing tobacco use treatment in outpatient psychiatry.

**Trial registration:**

Clinicaltrials.gov NCT04071795 (registered on August 28, 2019)

Contributions to the literature

- The majority of tobacco treatment implementation research has taken place in primary care. In contrast, this study will test strategies to improve tobacco treatment guideline adherence in the outpatient psychiatry setting.
- This study will be one of the first randomized controlled trials to evaluate the impact of a behavioral economics-driven strategy (opt-out clinical reminders) for implementing tobacco use treatment in an outpatient setting.
- This study will produce rich qualitative data assessing psychiatrist perceptions of—and other barriers/facilitators toward implementing—an opt-out tobacco treatment system.

## Introduction

Smoking is the leading preventable cause of death in the USA, responsible for over 440,000 deaths per year [1]. People with mental health diagnoses have rates of smoking that are two to four times higher than those found in the general population [2], and they smoke more heavily in terms of the number of cigarettes smoked per day and a longer draw per cigarette [3]. This causes considerable health consequences for this already vulnerable population. People with serious mental illnesses in particular die on average 25 years earlier than the general population, and 60% of this excess mortality risk is due to smoking-related illnesses [4].

Several effective tobacco treatments are available for smokers with and without a mental health diagnosis. The US Public Health Service (PHS) and the American Psychiatric Association (APA) practice guidelines for the treatment of tobacco use include five nicotine replacement medications (NRT) and two non-nicotine medications (bupropion and varenicline) [5, 6]. The PHS guidelines further recommend the combination of medications with behavioral therapy to produce the highest abstinence rates. Busy physicians who are unable to provide cessation counseling themselves can follow a brief 3As approach to providing tobacco treatment to mental health patients by *asking* patients about tobacco use, *advising* them to quit, and *assisting* them with quitting by prescribing cessation medications and referring them to a counseling program. However, even brief 3As approaches to tobacco treatment are not regularly implemented in mental health treatment settings, leaving smokers with psychiatric conditions under-screened and under-treated [7, 8].

Multiple barriers exist to increasing tobacco treatment in psychiatry which have not been adequately addressed in prior research. Mental health providers often view tobacco cessation as a low priority for their patients [9, 10], and many psychiatrists receive no training in tobacco cessation treatment in medical school or residency [10]. In a prior study, we implemented a telephone care program for smokers in six Veterans Health Administration (VA) mental health clinics [11] and conducted semi-structured interviews with mental health providers to understand the barriers toward referring patients to the program. These discussions revealed treatment barriers at multiple levels, including low organizational prioritization of tobacco control in psychiatry, lack of clarification for psychiatrists about their role in treating tobacco, lack of training and comfort among psychiatrists in treating tobacco, provider attitudes that smoking may benefit their patients or cessation may be harmful, and lack of treatment engagement by patients. Although some work has been done to improve tobacco training for psychiatrists [12], there is a paucity of research on how to best implement tobacco treatment in mental health care.

Current tobacco treatment systems may perpetuate barriers to tobacco treatment for vulnerable populations. Health care systems commonly use a “no treatment” default for tobacco, such that providers must actively choose (opt-in) to treat their patients who express interest in quitting and patients must actively opt to receive treatment. A failure to act by either provider or patient results in a failure to treat. *Default bias theory* and experimental evidence within the field of behavioral economics posit that humans have a bias to accept customary (*status quo*) or default options even in the presence of superior alternatives [13, 14]. Thus, in settings and populations for which tobacco treatment is uncommon or discouraged (such as psychiatry visits), an opt-in treatment approach may actually reinforce the status quo to not treat. In recognizing that opt-in treatment approaches can introduce or reinforce systematic barriers to treatment, there has been a call in the literature to change tobacco treatment within health care settings to an opt-out system, where tobacco treatment is defaulted (i.e., automatically initiated) unless the provider or patient actively declines [15]. Research has shown that restructuring default options can significantly affect health-related choices and behavior [16]. Opt-out systems have been successful at modifying employee retirement plan contributions [17] and at dramatically improving rates of organ donation and HIV screening [18], and preliminary evidence from an observational study suggests opt-out systems may increase the rate of tobacco treatment referrals in maternity clinics [19]. Thus far, this approach has not been tested as a means to implement tobacco treatment in a psychiatric setting.

## Methods

### Study aims

We aim to conduct a pilot implementation study with the following aims:

1. Estimate the effect of an opt-out versus opt-in tobacco treatment system on the proportion of mental health patients who are treated for tobacco use by their psychiatrist.
2. Assess provider fidelity to the opt-out system, provider perceptions of the opt-out system, and barriers and facilitators to implementation of the opt-out system.
3. Estimate the effect of the opt-out versus opt-in tobacco treatment system on the use of cessation treatment and abstinence among mental health patients who smoke.

### Study design

**Fig. 1:**
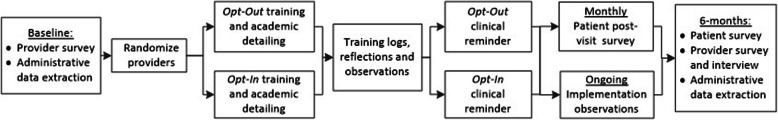
Overview of the methods and study design

Figure 1 displays an overview of our methods and study design. We will use a mixed-methods, two-arm cluster-randomized study design. Because the study is targeting provider behavior change, we will randomize at the provider level.

### Site

This study will take place at the VA New York Harbor Healthcare System (NYHHS), which serves primarily low-income veterans in New York City and surrounding areas. Approximately 60% of NYHHS patients are Caucasian, 31% are African American, 17% are Hispanic, and 61% have a high school education or less. The VA NYHHS receives approximately 14,000 mental health clinic visits each year, and 40% of mental health patients have documentation in the electronic health record (EHR) of current smoking. The VA NYHHS has pharmacotherapy and behavioral counseling available for all smokers.

### Conceptual framework

The overall study approach is guided by Proctor’s framework for implementation research and the Consolidated Framework for Implementation Research (CFIR) [20]. The Proctor framework includes three main processes to implementation research: (1) the selection of an evidence-based practice (EBP), (2) the development of strategies to implement the EBP, and (3) outcome measurement, which includes implementation outcomes, service outcomes, and client/patient outcomes. The CFIR identifies five main domains that can influence implementation outcomes: (1) an organization’s inner setting, (2) an organization’s outer setting, (3) the characteristics of the intervention, (4) the perceptions and characteristics of individuals who interact with the intervention, and (5) the implementation process.

**Fig. 2:**
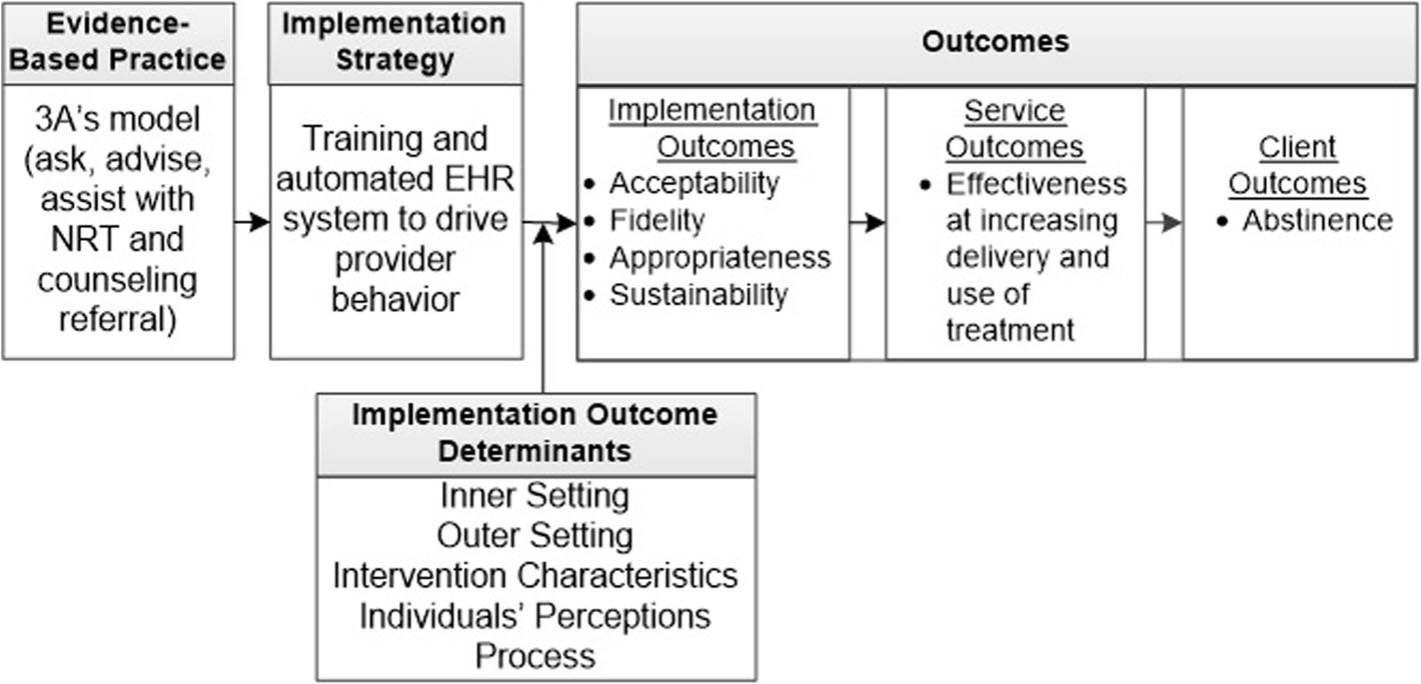
Conceptual framework for the study approach

Figure 2 displays our study approach within these frameworks. The study is designed to improve psychiatrist adherence to an evidence-based 3As model of tobacco treatment—*asking* about use, *advising* to quit, and *assisting* by prescribing NRT and referring to counseling. We will test two strategies for increasing psychiatrist adherence to this approach that will combine provider training with EHR system change to drive provider behavior. During aim 2, we will measure the implementation outcomes such as fidelity, acceptability, acceptability, and perceived sustainability of the implementation interventions, as moderated by CFIR domains. During aim 1, we will measure the effectiveness of the implementation interventions on provider perceptions of the opt-out system and barriers and facilitators to implementation of the opt-out system. During aim 3, we will measure the impact of the implementation interventions on patient use of treatment and abstinence.

### Implementation strategy framework

**Fig. 3:**
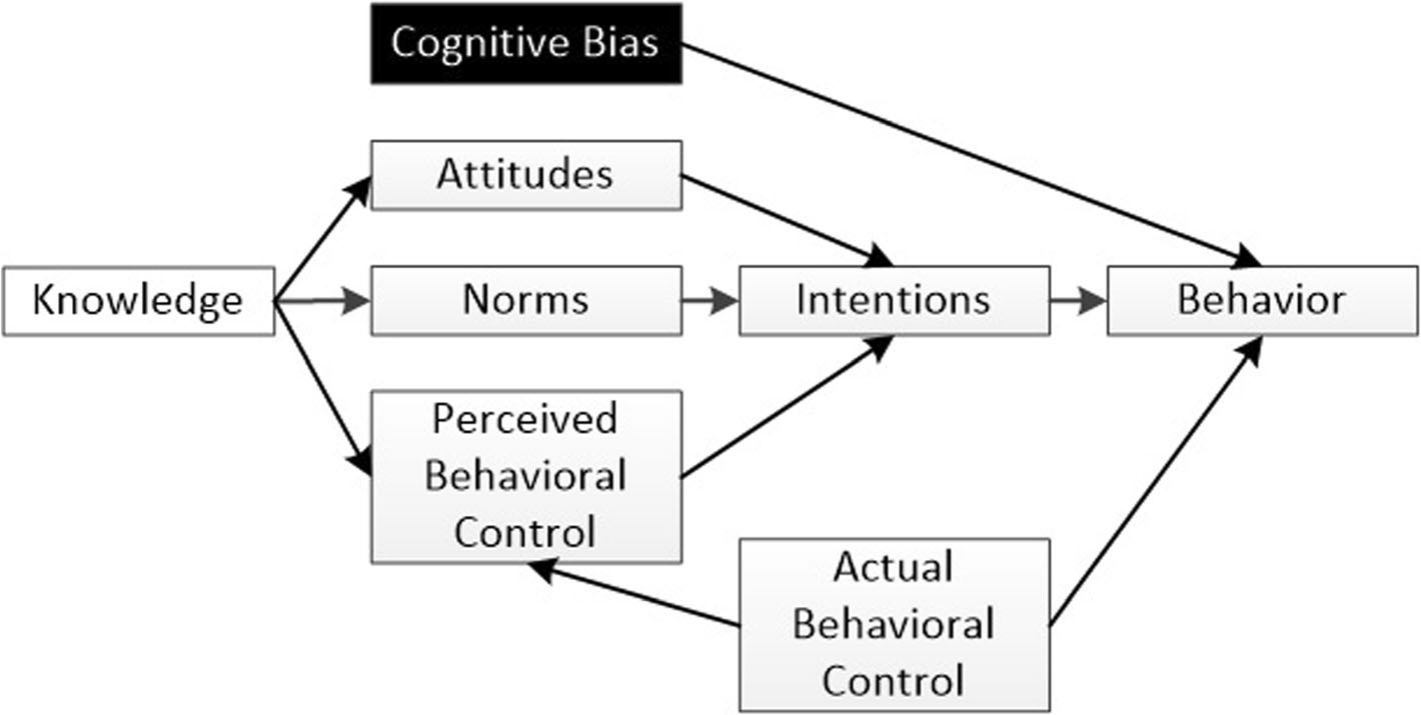
The theoretical framework that guided the design of the study’s implementation interventions

Figure 3 displays the theoretical framework that guided the design of the study’s implementation interventions, which combines the psychological Theory of Planned Behavior and the Default Bias Theory from the field of behavioral economics [21]. The Theory of Planned Behavior (TPB; shaded boxes in Fig. 2) posits that behavioral intentions are the most proximal determinant of behavior, and there are three primary antecedents of intentions: (1) attitudes/beliefs about a behavior, (2) perceived social norms about the behavior, and (3) perceived control over performing the behavior. The TPB also suggests that one’s actual behavioral control influences perceived control and directly influences behavior. The TBP has been shown to predict health care provider behavior in prior research [22], and the literature and our prior work have identified tobacco treatment barriers within psychiatry that align with the constructs of the TBP. For example, psychiatrists report that lack of training (knowledge) leads to low self-efficacy and comfort (perceived behavioral control) in treating tobacco and in working with patients who are resistant to treatment and that limited time (actual behavioral control) and lack of prioritization of tobacco among leaders and peers (social norms) limits their provision of tobacco treatment [10]. Provider training programs and traditional (opt-in) clinical reminder systems are designed to overcome such barriers that align with the TBP.

However, the behavioral economic theory posits that cognitive biases (black box in Fig. 3) lead directly to human choice and behavior, independent of one’s beliefs or intentions about a behavior [13, 14]. One such bias is the *default bias* (or *status quo bias*), which causes humans to select customary or default options even in the presence of superior or healthier alternatives. Opt-out systems take advantage of these cognitive biases by making the desired behavior (in our case, tobacco screening and treatment) the default.

### Implementation strategies

Table 1 outlines the components of the study’s implementation strategies and the constructs from the guiding theoretical framework (Fig. 3) that the components are designed to target.

**Table 1:** Components of the study’s implementation strategies and the constructs from the guiding theoretical framework

Strategy component	Targeted barriers
Both arms: Psychiatrist training and academic detailing	• Lack of provider knowledge about tobacco treatment • Low provider perceived behavioral control in treating patients for smoking and dealing with resistant patients • Negative attitudes and subjective norms toward the treatment of tobacco
Arm 1: Opt-in clinical reminder	• Low provider perceived behavioral control in tobacco treatment • Low organizational prioritization (norms) of tobacco treatment
Arm 2: Opt-out clinical reminder	• Cognitive bias to accept the default treatment • Low provider perceived behavioral control in tobacco treatment • Low prioritization (norms) of tobacco treatment • Limited time to screen and treat (actual behavioral control)

#### Both arms: psychiatrist training and academic detailing

Since psychiatrists cite the lack of training as a barrier to providing smoking cessation treatment, both arms will receive a 1-h training in helping their patients quit smoking [23]. The training will be adapted from the evidence-based *Psychiatry Rx for Change* training program for psychiatry residents [24]. We will also include topics that emerged as treatment barriers or facilitators during our previous qualitative interviews with psychiatrists, such as linking cessation to improved mental health treatment outcomes. To reinforce provider knowledge and self-efficacy in treating mental health patients for smoking, we will also implement academic detailing for providers in both arms. Academic detailing has consistently shown improvements in provider behavior [25]. Two study investigators will make a brief outreach visit to each psychiatrist, at least one of whom will be a physician to answer any questions about cessation medications. The investigators will follow the seven steps recommended by the National Resource Center for Academic Detailing [26]: (1) the introduction, (2) needs assessment, (3) key messages/features/benefits, (4) understanding barriers and enablers, (5) identifying and handling objections, (6) summary, and (7) close. The key message (step 3) will include the demonstration of the clinical reminder, review of the evidence for smoking cessation medications and how to prescribe them, and review of the role of the facility Health Promotion staff and how to contact them. Detailing will occur after randomization, so each psychiatrist will receive a detailing visit on the specific intervention to which he or she will be exposed.

#### Arm 1: Opt-in clinical reminder

To increase actual and perceived behavior control and to increase perceived prioritization (disciplinary norms) of the treatment of psychiatric patients who smoke, we will implement a tobacco clinical reminder embedded within the EHR. Tobacco use clinical reminders are the best practice recommended by the PHS tobacco treatment guidelines and are routinely tested and used to address tobacco in primary care settings [5]. All VA facilities currently use clinical reminders, which are adapted locally and can be adapted for specific individuals or groups of providers. The reminder will guide providers through each step of the 3As approach:

1. *Ask and advise*—Providers will be prompted to ask their patients if they currently use tobacco and advise the patient to quit and to use treatment if interested in quitting.
2. *Assist: medications*—Providers will be able to order cessation medications by clicking a box associated with an order template embedded in the reminder. The VA already has pre-set ordering templates for NRT (patch, gum, and lozenge), bupropion, and varenicline.
3. *Assist: referral to counseling*—Providers will be able to refer patients to the local cessation counseling program by clicking a consult box embedded in the reminder. The consult will be sent to the facility’s local cessation program.

#### Arm 2: Opt-out clinical reminder

For arm 2, we will directly change the treatment status quo by implementing a clinical reminder that automatically initiates an order for NRT and referral to the cessation program at the time a smoker is identified. The psychiatrist will need to actively cancel the NRT and counseling orders in order to opt the patient out of treatment. The reminder will include the following domains:

1. *Ask and advise*—Providers will be prompted to ask their patients if they currently use tobacco. Psychiatrists will be prompted to advise patients that the VA’s goal is to help all patients quit by prescribing NRT and referring them to tobacco cessation coaching.
2. *Assist: automatic medications*—The VA has pre-set ordering templates for NRT, bupropion, and varenicline. For smoking patients, the psychiatrist will receive an alert that an order for combination NRT (patch plus gum) will be placed unless the provider cancels the order by clicking a box within the reminder.
3. *Assist: automatic referral to counseling*—For smoking patients, the reminder will also automatically generate an electronic consult to the local smoking cessation program (described above). The reminder will include a box to check if the psychiatrist does not want the coordinator to follow up with the patient.

### Provider recruitment

One month before the implementation of the provider training and reminders, all psychiatrists in the facility will be notified of the study at a required staff meeting. Psychiatrists will have the option at the meeting to ask questions and provide written or verbal opt-out of study participation. Psychiatrists will also have the opportunity to ask questions one-on-one with the study investigators before deciding whether to participate. Psychiatrist turnover is low at the facility; however, during recruitment, we will ask the psychiatrists if they plan to leave in the next 6 months and only enroll those who have no plans to leave. We anticipate that 20 of 24 psychiatrists at the VA NYHHS will participate.

### Provider randomization

Psychiatrists who do not opt-out of participation will be randomized to one of the two study arms, stratified by site, supervised by the study’s statistician. Psychiatrists who opt-out of study participation will receive the opt-in clinical reminder as a part of their routine care, but their performance will not be included in the study analyses.

### Outcomes

The study’s primary outcomes are (1) the percent of smokers prescribed a cessation medication and (2) the percent of smokers referred to cessation counseling. Secondary outcomes will include patient use of cessation treatment and self-reported 7-day abstinence at 6 months. We will also measure the intervention fidelity and provider perceptions of the intervention components.

## Data sources and measures

### Aim 1: Cessation prescriptions and treatment referrals

Table 2 outlines our measures, data sources, and data collection schedule for the study. Our assessment plan for aim 1 is to use the VA administrative data to estimate and compare the effect of the opt-out versus opt-in treatment systems on the percent of all smokers treated for tobacco use by their psychiatrist during the study’s intervention period. The VA uses a fully EHR system that documents diagnostic and procedural data from all outpatient and inpatient encounters. The VA’s Informatics and Computing Infrastructure (VINCI) allows VA-affiliated researchers to query encounter data and has data analysts available to assist the investigators with data selection. We will work with VINCI programmers to identify all patients seen by a participating psychiatrist in the 6 months before and after implementation of the clinical reminder. We will then ask the VINCI programmers to calculate the percent of these patients who were screened for tobacco use by the psychiatrist, and then among smoking patients, the percent prescribed at least one cessation medication and the percent who were referred to the local cessation program.

**Table 2:** Measures, data sources, and data collection schedule for the study

Measures	Data source	Timing	Aim
Primary outcomes			
Proportion of smokers prescribed cessation medication	EHR	6 months pre/post-implementation	1
Proportion of smokers referred for counseling	EHR	6 months pre/post-implementation	1
Secondary outcomes			
Patient use of cessation treatment	Patient post-visit survey and follow-up survey	6 months pre/post-visit	3
Patient self-reported 7-day abstinence	Patient follow-up survey	6 months post-visit	3
Provider perceptions			
Provider perceptions of the intervention	Training observations Provider interviews	Training period 6 months post-implementation	2
Provider attitudes toward treatment, self-efficacy toward treatment, treatment norms, motivation and intention to treat	Provider survey	Baseline and 6 months post-implementation	2
Implementation barriers and facilitators			
Barriers/facilitators toward implementation of the intervention components	Provider interviews Observation logs	6 months post-implementation Intervention period	2
Implementation fidelity			
Provider training and detailing fidelity Provider delivery of 3As	Training logs Patient post-visit surveys	Training period Monthly during the intervention period	2
Other measures			
Patient characteristics: psychiatric diagnosis, utilization, sociodemographics, smoking history, quitting self-efficacy, attitudes toward treatment, motivation to quit	Patient post-visit survey	Post-visit	3

### Aim 2: Fidelity, provider perceptions, and implementation barriers and facilitators

#### Fidelity

##### Training logs

We will log all training activities to capture the proportion of participating psychiatrists who attended the training sessions and received an academic detailing visit, and the content and length of the academic detailing visits.

##### Post-visit surveys

Once a month during the 6-month intervention period, research assistants blinded to the group assignment will survey a random sample of patients seen by a participating psychiatrist within 24 h of their visit to assess provider fidelity to the 3As approach. To identify and recruit patients for the surveys, we will use the EHR to identify a list of patients seen by a participating psychiatrist on the day we run the EHR query. We will take a random selection of 5-10 male patients (depending on the response rate) and all female patients (to increase the representativeness of women) per psychiatrist at each monthly assessment point to reach out to for a survey. We will make 2 attempts over 24 h to reach the patient by phone to explain the study and obtain verbal consent. We aim to complete post-visit surveys with 20 patients per psychiatrist over the intervention period (*N* = 200/arm). Participants will receive $10 for completing the survey.

### Provider perceptions and implementation barriers/facilitators

#### Observations

During the group training sessions, the study coordinator will take notes and the trainers will complete reflection memos after each session which capture the psychiatrists’ reactions to the training content, questions asked, and any group discussion. The coordinator will also observe each academic detailing session and document psychiatrist reactions, comments, and questions. All study meeting minutes (excluding confidential information) will be analyzed qualitatively for themes related to provider perceptions and barriers/facilitators.

#### Provider survey

We will use a repeated measures design to conduct a survey with participating psychiatrists at baseline and 6 months assessing their attitudes, beliefs, motivations, and intentions to treat tobacco. We will invite providers to participate in the survey by sending an email to their VA email address. This email will include all elements of informed consent and a link to complete an online survey through the VA’s secure REDCap system [27]. We will send two reminders to non-respondents. Providers will be paid $10 for the completion of each survey. The survey will assess the following: attitudes toward the opt-in or opt-out reminder using the Evidence-Based Practice Attitude Scale (EBPAS) [28, 29], perceived level of control, subjective disciplinary norms and intentions to help their patients quit smoking using questions adapted from the Determinants of Implementation Behavior Questionnaire (DIBQ) [30], and intrinsic and extrinsic motivations to treat their patients for tobacco using items from the Treatment Self-Regulation Questionnaire [31]

#### Provider interview

Guided by the Proctor and CFIR models, we will conduct semi-structured interviews with 12–14 psychiatrists (6–7 per arm) assessing their views on the appropriateness, acceptability, and sustainability of the intervention components, as well as how the clinic’s inner setting (e.g., culture, norms, workflow compatibility with the intervention), outer setting (e.g., VA policies, psychiatric professional associations), psychiatrist characteristics (e.g., beliefs about the intervention), and the implementation process (e.g., how providers were informed, who championed the intervention) may impact their views on appropriateness, acceptability, and sustainability. We will also ask the psychiatrists for their insights into the challenges and successes encountered during their participation in the intervention components. We will recruit psychiatrists for interviews using sign-up sheets and by institutional email invitations. A trained interviewer will follow an interview guide with a set of pre-specified questions and follow-up probes. All interviews will be audio-taped. Psychiatrists will be paid $20 for completing an interview.

#### Aim 3: Patient use of cessation treatment and self-reported abstinence

We will assess patient use of cessation treatment and tobacco abstinence 6 months after seeing a participating psychiatrist. For this aim, we will conduct a follow-up telephone survey with the 400 cluster-sampled patients who completed a post-visit survey during the intervention period. The follow-up survey will ask patients to indicate whether they used a list of tobacco treatments in the prior 6 months, including all FDA-approved cessation medications, in-person cessation counseling, telephone cessation counseling, and a mobile texting cessation service. Consistent with the guidelines for measuring abstinence in pragmatic trials, the survey will also assess 7-day point prevalence abstinence [32]. We will make up to 10 attempts at different times of the day and month to reach patients by phone for a follow-up survey. Telephone non-respondents will be sent a survey in the mail with a pre-paid return envelope.

#### Other measures: patient characteristics

Our patient surveys will collect additional information, including sociodemographics (age, gender, marital status, race/ethnicity, income), attitudes toward tobacco treatment using the Attitudes Toward Nicotine Replacement Therapy Scale [33] adapted to ask about NRT and counseling, quitting self-efficacy using the Smoking Self-Efficacy Questionnaire [34], motivations to quit using a 0–10 scale, and smoking status and history using questions from the California Tobacco Survey [35].

### Analysis

We will first summarize the survey and administrative data using descriptive statistics (means, medians, standard deviations, frequency distributions, and graphical displays) to characterize providers and patients treated by the providers in the two intervention arms.

#### Aim 1

Based on the administrative data, we will categorize the proportion of smokers seen by a participating psychiatrist in each group who (1) received a cessation medication prescription from the psychiatrist or (2) referred to a cessation counseling by the psychiatrist. We will use generalized linear mixed effect models (GLMMs) with random effects for the clusters to compare these screening and treatment rates between the groups. For all analyses, sensitivity analyses will be used to evaluate the impact on results from missing data and subject dropout. Specifically, missingness will be handled by creating a separate category, or removed by multiple imputation (MI).

#### Aim 2

For intervention fidelity, we will use descriptive statistics to summarize the patient post-visit survey data to calculate the proportion of patients seen by a participating psychiatrist in each study arm who were asked about smoking, offered medications, and offered counseling referral, and (among those offered) the proportion of patients who accepted the medications and referral. We will also summarize training fidelity logs (the proportion of psychiatrists who attended the trainings and received an academic detailing session).

To assess provider perceptions quantitatively, we will summarize the provider survey data using descriptive statistics (means, standard deviations) to understand the providers’ attitudes and beliefs regarding treating their patients for tobacco. For our qualitative data analysis (interviews, observations, meeting minutes), we will use a three-step coding process for each data source. First, two investigators will individually read a sub-sample of data (e.g., three interviews) to identify preliminary inductive codes, then meet to achieve consensus on coding the sub-sample and create the first draft of a codebook. Second, the investigators will individually apply the codebook to a second sub-sample of data and meet to achieve coding consensus on the second sub-sample to create the final codebook for the data source. Third, once all data are coded, the investigators will meet to complete more focused coding to identify code clusters, relationships among codes, and common themes. Once all data sources are coded, we will also use group consensus meetings to look for themes across the main data sources.

#### Aim 3

We will use a similar analytic approach as in aim 1. We will first categorize each patient as (1) having achieved or not achieved 7-day abstinence 6 months after their psychiatry visit and (2) having used or not used any type of cessation treatment in the 6 months after their visit. This will estimate the use of treatment and abstinence rates for each study arm. We will use GLMMs to compare these two outcomes between the groups. Sensitivity analyses will be used to evaluate the impact on results from missing data and subject dropout. An ITT approach will be compared with the complete case-only method, but our primary analysis will be the complete case analysis, as the North American Quitline Consortium has found this approach to be more accurate in representing true quit rates and recommends the use of this calculation [36], as do other reviews [37].

#### Sample size and power

We aim to enroll all practicing psychiatrists at our study site but have conservatively estimated that 20 will enroll. We calculated the level of power this will provide us to find a significant group effect on the proportion of patients prescribed a cessation medication by their psychiatrist (primary outcome) at all during the intervention period. Our power calculation varied the intraclass correlation (ICC) of 0.05–0.15 based on a cluster-randomized trial of preventive care in primary care practices [38]. We estimate that 10% of patients in arm 1 will receive a prescription [8]. With the smallest ICC of 0.05, a type I error of 5%, and 80% power, 20 clusters (psychiatrists) will provide us with enough power to detect a 21% or greater prescription rate in arm 2. With the largest ICC of 0.15, a type I error of 5%, and 80% power, 20 clusters will provide us with enough power to detect a 29% or greater prescription rate in arm 2.

## Discussion

Mental health patients smoke at high rates but rarely receive treatment. Traditional opt-in treatment systems may reinforce multi-level barriers to treating mental health patients for tobacco use. This pilot study will be testing an opt-out system for implementing a 3As tobacco treatment approach in outpatient psychiatry. This pilot study will produce important data on the potential of opt-out systems to overcome barriers in implementing tobacco use treatment in outpatient psychiatry.

There are some potential limitations to this study. First, this study is taking place in only one VA site and therefore may have limited generalizability to other settings. However, conducting this study in a VA site allows us to conduct this preliminary work in highly cost-efficient manner using the VA’s rich informatics infrastructure and allows us to avoid organizational heterogeneity that may dilute effects in a pilot study. Second, there may be a provider-level contamination between the study arms. Providers in the opt-out arm may change their attitudes and behavior and convince their colleagues in the opt-in arm to follow. However, in practice, it is difficult to change provider behavior through colleague discussions alone. Third, there is a risk of patient contamination (seeing more than one psychiatrist randomized to different arms during the intervention period). However, patients almost always see the same psychiatrist at each visit, and we will use the VA’s EHR data to track and account for contamination in our analyses.

Despite these limitations, this study will produce important data on the potential of opt-out systems to overcome barriers in implementing tobacco use treatment in outpatient psychiatry.

## Data Availability

The datasets used and analyzed during the study are available from the corresponding author on reasonable request.

## Abbreviations

APA: American Psychiatric Association
CFIR: Consolidated Framework for Implementation Research
DIBQ: Determinants of Implementation Behavior Questionnaire
EBP: Evidence-based practice
EBPAS: Evidence-Based Practice Attitude Scale
EHR: Electronic health record
FDA: US Food and Drug Administration
GLMMS: Generalized linear mixed effect models
ICC: Intraclass correlation
ITT: Intent-to-treat
MI: Multiple imputation
NRT: Nicotine replacement therapy
NYHHS: New York Harbor Healthcare System
PHS: US Public Health Service
TPB: Theory of Planned Behavior
VA: Veterans Health Administration
VINCI: VA Informatics and Computing Infrastructure
